# Updated checklist of the Michigan (USA) caddisflies, with regional and habitat affinities

**DOI:** 10.3897/zookeys.730.21776

**Published:** 2018-01-17

**Authors:** David C. Houghton, R. Edward DeWalt, Angelica J. Pytel, Constance M. Brandin, Sarah E. Rogers, David E. Ruiter, Ethan Bright, Patrick L. Hudson, Brian J. Armitage

**Affiliations:** 1 Department of Biology, Hillsdale College, 33 East College Street, Hillsdale, MI 49242, USA; 2 Illinois Natural History Survey, 1816 South Oak Street, Champaign IL 61820, USA; 3 235 SW Central Avenue, Grants Pass, OR 97526, USA; 4 Museum of Zoology, University of Michigan, Ann Arbor, MI 48103, USA; 5 Great Lakes Science Center, US Geological Survey, 1451 Green Road, Ann Arbor, MI 48105, USA; 6 Instituto Conmemorativo Gorgas de Estudio de la Salud, Ave. Justo Arosemena y Calle 35, Apartado Postal No 0816-02593, Ciudad de Panamá, Republic of Panamá

**Keywords:** Michigan, Trichoptera, caddisfly, checklist, species, diversity

## Abstract

Based on examination of ~180,000 specimens from 695 collections of 443 localities collected from the 1930s to 2015 we report 295 species of caddisflies from Michigan. Of these, 41 are reported from the state for the first time. Another 18 species previously reported from Michigan are listed as doubtful. The 11 most abundant species collectively represented over half of all specimens collected. Conversely, 80 species were known from <10 specimens, and 27 species from a single specimen. The Michigan fauna is similar to those of Minnesota and Ohio, adjacent states with comparable recent collecting effort. Regional and habitat affinities for each Michigan species are reported herein. Due to the high level of species discovery over the last few years, despite a >80-year collecting history, it is likely that additional species remain undiscovered in the state.

## Introduction

Despite the ecological importance of caddisflies in aquatic ecosystems and their utility in biological monitoring (Barbour et al. 1999), the faunas of the northcentral U.S. and southcentral Canada are not well known. Only the faunas of Illinois (Ross 1944), Minnesota (Houghton 2012), and Ohio (Armitage et al. 2011) have been extensively studied. For the remainder of the region, basic species checklists have been compiled for the Indiana (Waltz and McCafferty 1983), Manitoba (Flannagan and Flannagan 1982), Michigan (Leonard and Leonard 1949b), North Dakota (Harris et al. 1980), and Wisconsin (Longridge and Hilsenhoff 1973) caddisflies. All of these studies are >30 years old, and it is difficult to ascertain if changes to the fauna have occurred during the interim.

**Figure 1.**
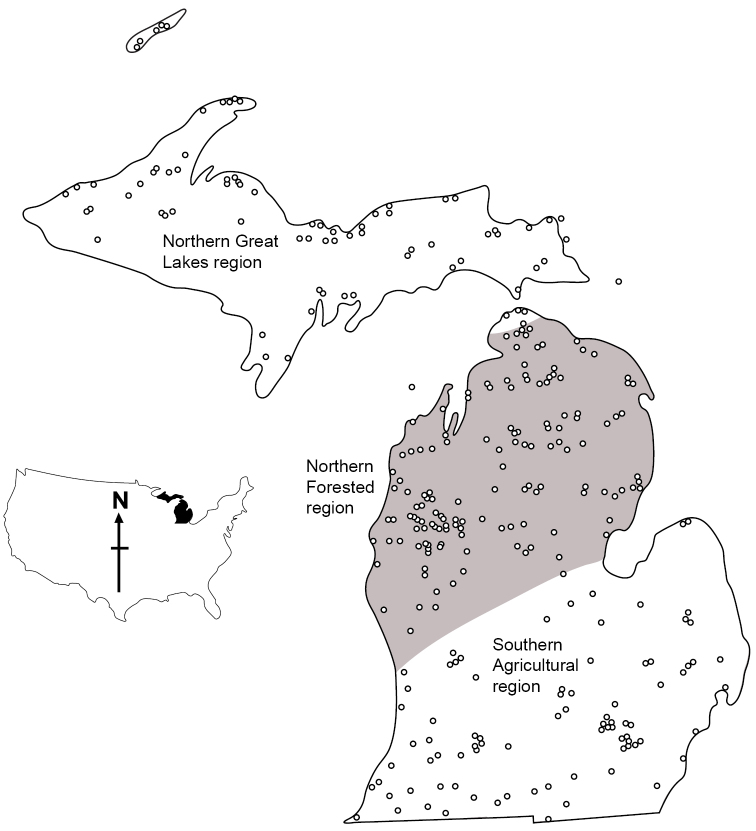
The determined caddisfly regions of Michigan (Houghton 2015), showing the collecting localities for this study.

The caddisflies of Michigan are known on a species level primarily from Leonard and Leonard’s (1949b) checklist. A compilation of known and suspected species is maintained by Bright (2017). The overall caddisfly distributions of the state have been divided into three distinct regions corresponding to the Northern Great Lakes, Northern Forested, and Southern Agricultural regions (Houghton 2015) (Figure 1). Many additional state records have been reported during the last 20 years (Table 1), but no comprehensive inventory of the state has occurred since the 1940s. Thus, the objectives of our study were to inventory the state and compile a comprehensive checklist of the Michigan fauna, and to relate this fauna to the three established caddisfly regions and different types of aquatic habitats.

**Table 1.** Past faunal studies of Michigan caddisflies, with resulting numbers of specimens, reported species, and new state records. Nearly all of the specimens associated with these studies were examined during the current study.

Reference	Region	Specimens	Species	State records
Ross (1938, 1941, 1944, 1946)	statewide	?	101	101
Leonard and Leonard (1949a)	statewide	?	N/A	7
Leonard and Leonard (1949b)	statewide	5,767	181	66
Ellis (1962)	Houghton Creek	?	85	0
Davis et al. (1991)	St. Clair/Detroit River	?	70	21
Houghton et al. (2011)	Manistee River watershed	26,000+	134	11
DeWalt and South (2015)	Isle Royale National Park	326	42	2
Houghton (2016)	Black River Ranch	38,248	117	3
Current paper	statewide	~180,000	291	41

## Materials and methods

We have been collecting caddisflies in Michigan since the 1990s, primarily using ultraviolet light traps for adults. Other adult collecting methods have included malaise trapping, sweep netting, and aspirating from riparian rocks and vegetation. Larval collection methods included kick-netting, hand-picking, and Hess sampling. Most adult collecting took place during June and July, the peak emergence period of caddisflies in central Michigan (Houghton et al. 2011). Additional collections of adults were made during May, August, and September to obtain early and late emerging species.

Collecting sites were chosen to yield a geographically representative sample, paying particular attention to unique habitats, such as intermittent streams, waterfalls, and forested wetlands. We collected from sites that appeared to be the least disturbed of their general area. Unique areas, such as the Huron and Porcupine Mountains in the northeastern Upper Peninsula, the Black River Ranch in the northern Lower Peninsula, Sleeping Bear Dunes National Park in the northwestern Lower Peninsula, and Sarah Jane’s Natural Area in the southern Lower Peninsula were sampled more intensively. Most notably, Fairbanks Creek, a pristine small stream in the northern Lower Peninsula, was sampled every week from May to October 2010–2014.

Specimens were identified using Houghton (2012) and more specific taxonomic treatments. Nomenclature follows that of Morse (2017). Most collected specimens and their respective locality data were databased using BIOTA software (Colwell 2007) and deposited in the Hillsdale College Insect Collection or the Illinois Natural History Survey. Some are in the personal collection of DER. Museum specimens from the University of Michigan were examined, especially records associated with Leonard and Leonard’s (1949b) checklist. Specimens referenced in Table 1 were also examined. Additional records were located in collections of Brigham Young University, the University of Minnesota, and Colorado State University. These specimens remain in their respective institutions.

## Results

Approximately 180,000 specimens from 695 collections of 443 Michigan localities from the 1930s to 2015 were examined during this study (Figure 1). From these specimens, we determined 295 total species, representing 20 families and 76 genera. Of these species, 41 are reported from Michigan for the first time and 204 are new since Leonard and Leonard’s (1949b) checklist. These species are presented in Table 2, along with their regional and habitat affinities and the depository of specimens. An additional 18 species previously reported from Michigan are listed as doubtful due to synonymy, misidentification, or our inability to locate a specimen (Table 3).

**Table 2.** The 295 caddisfly species confirmed from Michigan. Taxa are arranged alphabetically by family and genus. The number of species within each family is listed after each respective family. Species reported from Michigan for the first time are in boldface type. Collection data for each newly-reported species are included in Suppl. material 1. Spcs = total number of examined specimens, locs = total number of known localities. Dep. = museum with the largest number of deposited specimens. HCIC = Hillsdale College Insect Collection, INHS = Illinois Natural History Survey, UMMZ = University of Michigan Museum of Zoology, DER = personal collection of Dave Ruiter. NG = total specimens known from Northern Great Lakes caddisfly region, NF = total specimens from Northern Forested region, SA = total specimens from Southern Agricultural region (Figure 1). Lk = number of specimens known from lakes, SR = number of specimens from small (<4 m in width) rivers, MR = number of specimens from medium (4–15 m) rivers, LR = number of specimens from large (>15 m) rivers. Total number of specimens from the various habitats and regions may be less than the grand total of specimens for that species due to a lack of information about some collecting localities.

Taxon	spcs	locs	Dep.	NG	NF	SA	Lk	SR	MR	LR
APATANIIDAE (1)										
*Apatania zonella* (Zetterstedt, 1840)	49	4	HCIC	49	0	0	18	0	0	31
BRACHYCENTRIDAE (8)										
*Brachycentrus americanus* (Banks, 1899)	3547	81	HCIC	1278	2218	51	6	1094	2343	60
*B. fuliginosus* Walker, 1852	25	6	UMMZ	0	25	0	0	0	25	0
*B. incanus* Hagen, 1861	1	1	INHS	1	0	0	0	0	1	0
*B. lateralis* (Say, 1823)	69	4	UMMZ	0	69	0	0	0	69	0
*B. numerosus* (Say, 1823)	122	21	UMMZ	9	72	41	0	56	34	31
***Micrasema charonis* Banks, 1914**	5	2	INHS	0	2	3	0	0	2	3
*M. rusticum* (Hagen, 1868)	783	56	HCIC	24	722	37	4	97	669	13
*M. wataga* Ross, 1938	50	10	HCIC	40	10	0	0	3	44	3
DIPSEUDOPSIDAE (1)										
*Phylocentropus placidus* (Banks, 1905)	274	24	HCIC	235	17	21	71	86	104	3
GLOSSOSOMATIDAE (8)										
*Agapetus hessi* Leonard & Leonard, 1949	102	3	HCIC	0	102	0	0	0	102	0
*A. tomus* Ross, 1941	63	8	HCIC	27	0	36	0	12	49	0
*Glossosoma intermedium* Klapálek, 1892	16	9	HCIC	18	0	0	2	0	14	0
*G. lividum* (Hagen, 1861)	268	8	UMMZ	0	268	0	0	9	259	0
*G. nigrior* Banks, 1911	1796	68	HCIC	179	1575	42	22	1355	395	10
*Protoptila erotica* Ross, 1938	138	12	HCIC	4	130	4	1	0	63	74
*P. maculata* (Hagen, 1861)	76	10	HCIC	2	5	69	1	4	38	22
*P. tenebrosa* (Walker, 1852)	444	27	HCIC	223	220	1	6	197	223	18
GOERIDAE (1)										
*Goera stylata* Ross, 1938	2422	12	HCIC	30	2392	0	0	2361	58	3
HELICOPSYCHIDAE (1)										
*Helicopsyche borealis* (Hagen, 1861)	6957	114	HCIC	281	6347	310	1186	1703	3161	800
HYDROPSYCHIDAE (35)										
*Arctopsyche ladogensis* (Kolenati, 1859)	16	2	UMMZ	15	1	0	0	1	15	0
*Cheumatopsyche analis* (Banks, 1908)	1137	99	HCIC	334	485	318	80	263	625	53
*C. aphanta* Ross, 1938	38	2	HCIC	0	37	1	0	0	38	0
*C. campyla* Ross, 1938	6683	65	HCIC	55	257	6371	56	13	248	6312
*C. gracilis* (Banks, 1899)	1063	64	HCIC	248	804	11	2	33	912	114
*C. minuscula* (Banks, 1907)	2	1	HCIC	2	0	0	0	0	0	2
*C. oxa* Ross, 1938	1609	58	HCIC	69	1425	112	3	1077	516	8
*C. pasella* Ross, 1941	44	9	HCIC	6	37	1	2	1	41	0
*C. sordida* (Hagen, 1861)	7	4	HCIC	5	0	2	0	1	0	5
*C. speciosa* (Banks, 1904)	61	2	HCIC	0	0	61	0	0	0	58
*Diplectrona modesta* Banks, 1908	1106	9	HCIC	5	1096	5	0	1106	0	0
*Hydropsyche aerata* Ross, 1938	5	2	INHS	0	0	4	0	0	0	0
*H. alhedra* (Ross, 1939)	56	7	HCIC	15	40	1	0	5	49	2
*H. alternans* (Walker, 1852)	118	11	HCIC	115	2	1	57	2	0	54
***H. arinale* Ross, 1938**	1	1	INHS	1	0	0	0	0	1	0
*H. betteni* Ross, 1938	1262	67	HCIC	74	1041	147	13	932	266	33
*H. bronta* (Ross, 1938)	192	43	HCIC	37	96	59	1	17	141	33
*H. cheilonis* (Ross, 1938)	17	8	HCIC	0	2	14	0	0	0	4
*H. cuanis* Ross, 1938	21	4	INHS	0	0	21	0	7	8	6
*H. dicantha* Ross, 1938	11	7	HCIC	1	6	2	0	2	6	1
*H. frisoni* Ross, 1938	73	11	INHS	1	36	32	0	0	67	3
*H. incommoda* Hagen, 1861	130	14	HCIC	1	12	116	1	2	13	74
*H. leonardi* Ross, 1938	2	1	INHS	0	2	0	0	0	2	0
*H. morosa* (Hagen, 1861)	262	32	HCIC	62	162	39	10	18	165	61
*H. phalerata* Hagen, 1861	31	5	HCIC	0	4	27	0	0	0	23
***H. placoda* Ross, 1941**	1	1	HCIC	0	0	1	0	0	0	1
*H. scalaris* Hagen, 1861	3	3	INHS	0	0	2	0	0	0	0
*H. simulans* Ross, 1938	26	4	HCIC	0	22	4	3	0	3	16
*H. slossonae* (Banks, 1905)	1241	68	HCIC	137	1036	68	0	586	646	7
*H. sparna* (Ross, 1938)	2712	113	HCIC	425	2018	261	12	1253	1330	88
*H. vexa* (Ross, 1938)	12	6	HCIC	4	8	0	0	4	7	1
*H. walkeri* (Betten & Mosely, 1940)	65	13	HCIC	42	22	1	1	1	24	39
*Macrostemum zebratum* (Hagen, 1861)	533	15	HCIC	10	499	24	10	2	24	490
*Parapsyche apicalis* (Banks, 1908)	220	19	HCIC	62	252	6	1	274	45	0
*Potamyia flava* (Hagen, 1861)	119	16	HCIC	1	71	47	0	45	31	28
HYDROPTILIDAE (63)										
*Agraylea multipunctata* Curtis, 1834	4952	59	HCIC	127	927	3898	195	581	339	31
*Hydroptila ajax* Ross, 1938	27	3	HCIC	0	0	27	0	0	0	19
***H. albicornis* Hagen, 1861**	1	1	HCIC	1	0	0	0	0	1	0
*H. amoena* Ross, 1938	8	4	HCIC	1	7	0	2	0	6	0
***H. ampoda* Ross, 1941**	15	9	HCIC	15	0	0	0	0	15	0
***H. angusta* Ross, 1938**	45	2	HCIC	0	0	45	0	0	0	45
***H. antennopedia* Sykora & Harris, 1994**	111	9	HCIC	111	0	0	6	12	93	0
*H. armata* Ross, 1938	48	17	HCIC	6	13	29	2	2	40	1
*H. berneri* Ross, 1941	1	1	UMMZ	0	0	1	0	0	0	0
*H. calia* Denning, 1948	1	1	UMMZ	0	0	0	0	0	1	0
*H. consimilis* Morton, 1905	195	28	HCIC	31	140	24	0	62	129	4
***H. delineata* Morton, 1905**	1	1	HCIC	1	0	0	0	0	1	0
*H. grandiosa* Ross, 1938	20	9	HCIC	0	9	11	0	1	9	1
*H. hamata* Morton, 1905	154	27	HCIC	46	100	8	62	5	75	4
*H. jackmanni* Blickle, 1963	477	37	HCIC	168	270	39	4	191	278	0
*H. metoeca* Blickle & Morse, 1954	166	16	HCIC	1	165	0	3	80	82	1
***H. nicoli* Ross, 1941**	1	1	HCIC	1	0	0	0	0	1	0
*H. novicola* Blickle & Morse, 1954	1	1	HCIC	1	0	0	0	0	1	0
*H. perdita* Morton, 1905	11	6	HCIC	0	3	8	0	1	5	0
*H. quinola* Ross, 1947	3	2	HCIC	3	0	0	0	0	3	0
***H. salmo* Ross, 1941**	1	1	HCIC	1	0	0	0	0	1	0
***H. scolops* Ross, 1938**	18	2	UMMZ	0	16	2	0	0	16	0
*H. spatulata* Morton, 1905	9	5	HCIC	2	5	2	0	1	4	4
***H. tortosa* Ross, 1938**	7	1	HCIC	0	7	0	0	0	7	0
***H. tusculum* Ross, 1947**	1	1	HCIC	0	1	0	0	0	1	0
*H. valhalla* Denning, 1947	90	15	HCIC	88	2	0	0	2	87	1
*H. waubesiana* Betten, 1934	119	23	HCIC	7	36	76	8	17	51	3
*H. wyomia* Denning, 1948	23	5	HCIC	5	15	0	0	0	20	0
*H. xera* Ross, 1938	237	19	HCIC	189	51	0	2	1	235	2
***Ithytrichia clavata* Morton, 1905**	222	6	HCIC	1	214	7	139	0	75	8
*Leucotrichia pictipes* (Banks, 1911)	30	2	HCIC	15	15	0	0	0	21	9
*Mayatrichia ayama* Mosely, 1905	7	2	UMMZ	0	6	1	0	0	6	0
*Neotrichia halia* Denning, 1948	131	3	HCIC	131	0	0	0	0	5	126
***N. minutisimella* (Chambers, 1873)**	1	1	HCIC	1	0	0	0	0	0	1
***N. okopa* Ross, 1939**	9	1	INHS	9	0	0	0	0	9	0
*N. vibrans* Ross, 1938	1	1	HCIC	0	1	0	0	0	0	1
*Ochrotrichia arva* (Ross, 1941)	3	2	HCIC	0	3	0	0	2	1	0
*O. spinosa* (Ross, 1938)	220	11	HCIC	209	4	7	7	46	161	6
***O. riesi* Ross, 1944**	2	1	INHS	0	2	0	0	2	0	0
*O. tarsalis* (Hagen, 1861)	2	2	HCIC	1	0	1	0	0	2	0
*Orthotrichia aegerfasciella* (Chambers, 1873)	451	21	HCIC	1	64	386	38	5	54	5
*O. balduffi* Kingsolver & Ross, 1961	97	19	HCIC	11	42	44	25	2	36	4
*O. cristata* Morton, 1905	1813	49	HCIC	55	308	1450	163	71	187	0
*O. curta* Kingsolver & Ross, 1961	13	1	HCIC	13	0	0	13	0	0	0
*Oxyethira aeola* Ross, 1938	44	8	HCIC	0	44	0	0	5	39	0
*O. anabola* Blickle,1966	7	5	HCIC	5	2	0	0	1	4	2
***O. araya* Ross, 1941**	1	1	HCIC	1	0	0	0	0	1	0
*O. coercens* Morton, 1905	115	19	HCIC	7	101	7	4	29	77	0
*O. ecornuta* Morton, 1893	73	3	HCIC	2	71	0	73	0	0	0
*O. forcipata* Mosely, 1934	170	18	HCIC	9	21	140	11	5	20	0
*O. grisea* Betten, 1934	180	5	UMMZ	0	106	74	50	0	56	0
***O. itascae* Monson & Holzenthal, 1993**	4	4	HCIC	0	4	0	0	0	4	0
*O. michiganensis* Mosely, 1934	219	25	HCIC	120	99	0	3	32	183	1
*O. novasota* Ross, 1944	2	1	UMMZ	0	0	2	0	0	0	0
*O. obtatus* Denning, 1947	27	10	HCIC	4	15	8	12	0	5	3
*O. pallida* (Ross, 1904)	757	10	HCIC	0	9	748	4	1	28	0
*O. rivicola* Blickle & Morse, 1954	85	7	HCIC	2	83	0	1	59	25	0
*O. serrata* Ross, 1938	366	17	HCIC	6	319	41	315	0	11	0
***O. sida* Blickle & Morse, 1954**	10	5	HCIC	4	6	0	0	2	8	0
*O. verna* Ross, 1938	3	3	HCIC	1	1	1	0	1	0	0
*O. zeronia* Ross, 1941	73	12	HCIC	5	26	42	25	4	5	0
***Stactobiella delira* (Ross, 1938)**	27	4	HCIC	24	3	0	0	14	13	0
***S. palmata* (Ross, 1938)**	14	4	HCIC	5	9	0	0	0	5	9
LEPIDOSTOMATIDAE (10)										
*Lepidostoma bryanti* (Banks, 1908)	7129	38	HCIC	157	6907	65	7	6822	300	0
***L. carrolli* Flint, 1958**	1	1	HCIC	0	1	0	0	1	0	0
*L. cinereum* (Banks, 1914)	154	5	UMMZ	8	146	0	1	134	19	0
*L. costale* (Banks, 1914)	30	5	UMMZ	6	24	0	0	13	15	0
*L. griseum* (Banks, 1911)	406	8	HCIC	0	406	0	0	405	1	0
***L. liba* Ross, 1941**	1	1	INHS	0	1	0	0	1	0	0
*L. sackeni* (Banks, 1936)	14	7	HCIC	2	12	0	0	11	3	0
*L. togatum* (Hagen, 1861)	5623	87	HCIC	1167	4436	2	43	1022	4417	123
*L. unicolor* (Banks, 1911)	4	2	HCIC	4	0	0	0	0	4	0
*L. vernale* (Banks, 1897)	116	10	HCIC	1	115	0	0	112	1	3
LEPTOCERIDAE (46)										
*Ceraclea alagma* (Ross, 1938)	1058	34	HCIC	28	867	163	841	5	52	2
***C. albosticta* (Hagen, 1861)**	2	1	HCIC	0	2	0	2	0	0	0
***C. alces* (Ross, 1941)**	1	1	HCIC	0	1	0	0	0	1	0
***C. ancylus* (Vorhies, 1909)**	11	5	HCIC	10	0	1	1	0	9	1
*C. annulicornis* (Martynov, 1910)	1	1	HCIC	1	0	0	0	0	0	1
*C. arielles* (Denning, 1942)	3637	13	HCIC	515	3122	0	1	28	3607	1
*C. cancellata* (Betten, 1942)	163	31	HCIC	75	52	35	41	1	71	49
*C. diluta* (Hagen, 1861)	29	10	HCIC	10	8	10	22	0	5	1
*C. excisa* (Morton, 1904)	1	1	UMMZ	1	0	0	0	0	0	0
*C. flava* (Ross, 1904)	39	1	HCIC	39	0	0	0	0	0	39
*C. maculata* (Banks, 1899)	143	15	HCIC	39	68	36	11	1	100	31
*C. mentiea* (Walker, 1852)	1	1	INHS	0	0	1	0	0	0	1
*C. resurgens* (Walker, 1852)	7	4	HCIC	3	3	1	5	0	0	2
*C. tarsipunctata* (Vorhies, 1909)	1532	18	HCIC	113	1250	126	321	42	533	628
*C. transversa* (Hagen, 1861)	993	61	HCIC	311	666	14	53	17	866	53
*C. wetzeli* (Ross, 1941)	30	3	HCIC	26	4	0	0	0	4	26
*Leptocerus americanus* (Banks, 1899)	3037	85	HCIC	120	1010	1906	264	123	2139	365
*Mystacides interjecta* (Banks, 1914)	1067	52	HCIC	233	806	28	965	25	53	0
*M. sepulchralis* (Walker, 1852)	1774	102	HCIC	385	1299	89	1078	17	614	12
*Nectopsyche albida* (Walker, 1852)	2572	63	HCIC	21	2347	201	774	111	356	1278
*N. candida* (Hagen, 1861)	824	15	HCIC	4	92	728	90	0	350	383
*N. diarina* (Ross, 1944)	76	16	HCIC	9	55	12	20	2	48	1
*N. exquisita* (Walker, 1852)	226	19	HCIC	54	91	81	82	0	8	80
*N. pavida* (Hagen, 1861)	170	18	HCIC	43	125	2	90	1	77	1
*Oecetis avara* (Banks, 1895)	5654	37	HCIC	5321	269	69	76	2	437	5144
*O. cinerascens* (Hagen, 1861)	812	89	HCIC	199	465	148	494	66	144	8
*O. disjuncta* (Banks, 1920)	119	9	HCIC	28	90	1	0	1	116	2
***O. ditissa* Ross, 1966**	1	1	INHS	0	1	0	1	0	0	0
*O. houghtoni* Blahnik & Holzenthal, 2014	6	2	HCIC	4	2	0	6	0	0	0
*O. immobilis* (Hagen, 1861)	28	8	HCIC	5	23	0	21	3	3	0
*O. inconspicua* (Walker, 1852)	16220	168	HCIC	1383	12262	2550	8727	2928	2184	159
***O. nocturna* Ross, 1966**	2	2	HCIC	0	1	1	0	0	2	0
***O. ochracea* Curtis, 1825**	3	2	INHS	1	0	2	0	0	1	0
*O. osteni* Milne, 1934	444	55	HCIC	71	343	30	333	16	72	17
*O. persimilis* (Banks, 1907)	1422	68	HCIC	365	987	70	40	222	1085	72
*Setodes incertus* (Walker, 1852)	1543	23	HCIC	905	638	0	3	13	1371	156
*S. oligius* (Ross, 1938)	308	16	HCIC	0	262	46	180	2	79	8
*Triaenodes abus* Milne, 1935	125	14	HCIC	4	15	106	8	0	10	2
*T. baris* Ross, 1938	57	20	HCIC	8	43	6	2	29	18	1
*T. dipsius* Ross, 1938	98	17	HCIC	30	68	0	3	8	87	0
*T. ignitus* (Walker, 1852)	186	35	HCIC	9	131	46	3	13	159	11
*T. injustus* (Hagen, 1861)	535	56	HCIC	152	310	68	224	69	197	4
*T. marginatus* Sibley, 1926	334	42	HCIC	58	197	83	2	69	188	1
***T. melacus* Ross, 1947**	6	3	HCIC	0	0	6	4	2	0	0
*T. nox* Ross, 1941	107	26	HCIC	11	89	7	14	56	34	0
*T. tardus* Milne, 1934	1015	54	HCIC	58	399	557	23	165	288	5
LIMNEPHILIDAE (49)										
*Anabolia bimaculata* (Walker, 1852)	207	42	HCIC	72	114	10	57	21	108	1
*A. consocia* (Walker, 1852)	90	27	HCIC	14	64	12	3	12	61	1
*A. ozburni* Milne, 1935	254	5	UMMZ	0	23	231	0	0	23	0
*A. sordida* Hagen, 1861	9	6	INHS	1	6	2	4	0	4	0
*Asynarchus montanus* (Banks, 1907)	45	8	HCIC	2	15	28	3	1	12	0
*A. rossi* Leonard & Leonard, 1949	15	3	UMMZ	0	15	0	0	15	0	0
*Frenesia missa* (Milne, 1935)	159	13	UMMZ	1	156	2	1	77	77	0
*Glyphopsyche irrorata* (F., 1781)	7	4	HCIC	2	5	0	0	5	2	0
*Hesperophylax designatus* (Walker, 1852)	154	24	HCIC	17	126	11	1	119	37	0
*Hydatophylax argus* (Harris, 1869)	130	35	HCIC	6	119	2	1	71	54	0
*Ironoquia lyrata* (Ross, 1938)	4	2	HCIC	0	4	0	0	4	0	0
*I. parvula* (Banks, 1900)	4	2	INHS	4	0	0	2	0	2	0
*I. punctatissima* (Walker, 1852)	65	7	HCIC	0	26	39	0	25	0	0
*Lenarchus crassus* (Banks, 1920)	2	1	HCIC	2	0	0	0	1	0	0
*Leptophylax gracilis* Banks, 1900	11	7	UMMZ	0	6	5	0	1	0	1
*Limnephilus ademus* Ross, 1941	1	1	DER	1	0	0	0	0	0	0
***L. argenteus* Banks, 1914**	1	1	HCIC	1	0	0	0	0	1	0
*L. canadensis* Banks, 1808	7	6	UMMZ	5	2	0	0	1	5	0
*L. dispar* McLachlan, 1875	6	2	UMMZ	0	0	6	0	0	0	0
*L. externus* Hagen, 1861	3	2	UMMZ	0	3	0	0	0	3	0
***L. extractus* Walker, 1852**	1	1	INHS	1	0	0	0	0	0	0
*L. hyalinus* Hagen, 1861	1	1	HCIC	1	0	0	0	0	1	0
*L. indivisus* Walker, 1852	473	46	HCIC	2	116	351	12	65	32	6
*L. infernalis* (Banks, 1914)	15	5	UMMZ	14	0	0	14	0	0	0
***L. janus* Ross, 1938**	7	1	HCIC	7	0	0	7	0	0	0
*L. moestus* Banks, 1908	186	44	HCIC	63	91	32	1	59	70	3
*L. ornatus* Banks, 1907	97	31	HCIC	33	23	41	4	15	30	1
*L. parvulus* (Banks, 1905)	55	3	UMMZ	3	0	52	2	0	1	0
*L. perpusillus* Walker, 1852	25	2	UMMZ	0	0	25	0	0	0	0
*L. rhombicus* (L., 1758)	62	13	HCIC	14	47	1	2	11	49	0
*L. sackeni* Banks, 1930	4	4	UMMZ	2	1	2	1	0	2	1
*L. samoedus* McLachlan, 1880	3	2	DER	0	0	0	2	0	0	0
*L. secludens* Banks, 1914	2	2	UMMZ	0	0	2	0	0	0	0
*L. sericeus* (Say, 1824)	211	16	HCIC	12	195	4	8	25	171	0
*L. submonilifer* Walker, 1852	529	34	HCIC	19	105	405	0	71	32	0
*Nemotaulius hostilis* (Hagen, 1873)	45	7	HCIC	2	43	0	0	42	2	1
*Onocosmoecus unicolor* (Banks, 1897)	73	14	HCIC	45	28	0	0	14	59	0
*Phanocelia canadensis* (Banks, 1924)	4	1	UMMZ	0	0	4	0	0	0	0
*Platycentropus amicus* (Hagen, 1861)	15	4	HCIC	4	11	0	0	0	11	4
*P. radiatus* (Say, 1824)	386	68	HCIC	61	230	79	64	155	79	0
*P. indistinctus* (Walker, 1852)	1	1	HCIC	1	0	0	0	0	1	0
*Pseudostenophylax sparsus* (Banks, 1908)	20	10	HCIC	7	13	0	0	11	8	0
*Psychoglypha subborealis* Ross, 1944	5	2	UMMZ	0	5	0	0	3	2	0
*Pycnopsyche antica* (Walker, 1852)	2191	12	HCIC	0	2191	0	0	2165	5	0
*P. guttifera* (Walker, 1852)	1387	26	HCIC	23	1348	16	9	1309	50	0
*P. indiana* (Ross, 1938)	13	2	HCIC	0	1	12	0	0	13	0
*P. lepida* (Hagen, 1861)	236	38	HCIC	85	136	11	17	92	100	0
*P. scabripennis* (Rambur, 1842)	4	4	INHS	0	3	1	0	3	1	0
*P. subfasciata* (Say, 1828)	62	11	HCIC	28	3	30	27	0	0	0
MOLANNIDAE (5)										
*Molanna blenda* Sibley, 1926	563	29	HCIC	48	513	2	4	513	45	1
*M. flavicornis* Banks, 1914	67	12	HCIC	11	0	0	10	0	1	0
*M. tryphena* Betten, 1934	198	42	HCIC	111	67	15	2	33	156	0
*M. ulmerina* Navas, 1934	22	7	INHS	2	20	0	17	0	0	5
*M. uniophila* Vorhies, 1909	2027	65	HCIC	105	1850	68	1915	3	80	0
ODONTOCERIDAE (1)										
*Psilotreta indecisa* (Walker, 1852)	1	1	UMMZ	1	0	0	0	1	0	0
PHILOPOTAMIDAE (6)										
*Chimarra aterrima* Hagen, 1861	549	44	HCIC	136	396	14	45	137	323	16
*C. feria* (Ross, 1941)	213	8	HCIC	0	209	4	0	210	3	0
*C. obscura* (Walker, 1852)	4488	62	HCIC	69	1093	3326	65	136	3773	510
*C. socia* (Hagen, 1861)	8744	16	HCIC	8678	68	1	2	0	93	8646
*Dolophilodes distinctus* (Walker, 1852)	1343	73	HCIC	297	1043	1	10	750	581	0
*Wormaldia moesta* (Banks, 1914)	8	3	HCIC	8	0	0	0	0	8	0
PHRYGANEIDAE (18)										
*Agrypnia colorata* (Hagen, 1873)	3	3	UMMZ	1	1	0	1	0	1	0
*A. improba* (Hagen, 1873)	147	22	HCIC	130	17	0	46	2	99	0
*A. macdunnoughi* (Milne, 1931)	6	3	HCIC	2	0	0	0	0	2	0
*A. straminea* Hagen, 1873	18	7	INHS	18	0	0	18	0	0	0
*A. vestita* (Walker, 1852)	49	13	HCIC	2	33	14	5	29	4	0
*Banksiola crotchi* Banks, 1844	2219	92	HCIC	420	1644	135	352	1094	609	19
*B. dossuaria* (Say, 1828)	108	7	HCIC	0	108	0	0	106	1	1
*B. smithi* (Banks, 1914)	73	17	HCIC	35	21	16	28	0	27	0
***Beothukus complicatus* (Banks, 1924)**	2	2	?	2	0	0	2	0	0	0
*Fabria inornata* (Banks, 1907)	1	1	?	0	0	1	0	0	0	0
*Hagenella canadensis* (Banks, 1907)	50	10	HCIC	3	16	31	0	10	9	0
*Oligostomis ocelligera* (Walker, 1852)	10	1	UMMZ	0	0	0	0	0	0	0
*Phryganea cinerea* Walker, 1852	213	43	HCIC	101	101	2	48	62	89	4
*P. sayi* Milne, 1931	31	10	HCIC	0	27	4	1	24	4	0
*Ptilostomis angustipennis* Hagen, 1873	44	13	HCIC	1	36	7	3	33	1	0
*P. ocellifera* (Walker, 1852)	375	59	HCIC	79	252	44	42	173	126	3
*P. postica* (Walker, 1852)	7	6	HCIC	0	3	4	1	1	1	0
*P. semifasciata* (Say, 1828)	207	41	HCIC	131	48	22	14	23	161	0
POLYCENTROPODIDAE (28)										
*Cernotina spicata* Ross, 1938	135	11	HCIC	1	70	64	64	3	4	0
*Cyrnellus fraternus* (Banks, 1905)	45	8	HCIC	5	2	38	15	0	6	22
*Holocentropus flavus* Banks, 1908	75	14	HCIC	4	18	53	3	11	12	0
*H. interruptus* Banks, 1914	798	47	HCIC	47	246	505	156	95	63	2
*H. melanae* Ross, 1938	45	8	HCIC	2	17	26	16	0	3	0
***H. milaca* (Etnier, 1968)**	31	1	HCIC	0	31	0	31	0	0	0
***H. picicornis* (Stephens, 1836)**	20	2	HCIC	0	0	20	0	0	0	0
*Neureclipsis bimaculata* (L., 1758)	42	5	HCIC	36	5	1	4	0	28	9
*N. crepuscularis* (Walker, 1852)	276	59	HCIC	69	174	32	17	19	197	39
***N. piersoni* Frazer & Harris, 1991**	10	5	INHS	0	8	2	5	2	3	0
*N. validus* (Walker, 1852)	2	1	HCIC	2	0	0	2	0	0	0
*Nyctiophylax affinis* (Banks, 1897)	4982	83	HCIC	285	3496	186	3027	198	501	236
*N. moestus* Banks, 1911	160	17	HCIC	46	84	30	1	2	151	0
***N. serratus* Lago & Harris, 1985**	1	1	INHS	0	1	0	1	0	0	0
*Plectrocnemia albipuncta* Banks, 1930	40	14	HCIC	35	5	0	3	2	35	0
*P. aureola* Banks, 1930	862	24	HCIC	5	2	816	4	16	26	0
*P. cinerea* (Hagen, 1861)	1147	81	HCIC	88	778	276	684	37	117	4
*P. clinei* Milne, 1936	53	17	HCIC	12	41	0	10	31	12	0
*P. crassicornis* (Walker, 1852)	285	14	HCIC	2	20	163	2	2	19	0
***P. icula* (Ross, 1941)**	10	5	HCIC	9	1	0	0	0	10	0
*P. nascotia* (Ross, 1941)	28	2	HCIC	0	0	28	0	0	28	0
*P. remota* (Banks, 1911)	230	29	HCIC	7	51	171	4	30	25	0
*P. sabulosa* (Leonard & Leonard, 1949)	3	1	UMMZ	0	3	0	0	0	0	0
*P. weedi* (Blickle & Morse, 1955)	4	4	HCIC	0	2	2	0	1	1	0
*Polycentropus centralis* Banks, 1914	33	2	HCIC	33	0	0	0	0	33	0
*P. confusus* Hagen, 1861	27	11	HCIC	7	16	0	0	0	21	1
*P. pentus* Ross, 1941	678	63	HCIC	101	541	36	8	354	296	13
*P. timesis* (Denning, 1948)	15	4	HCIC	0	15	0	0	15	0	0
PSYCHOMYIIDAE (2)										
*Lype diversa* (Banks, 1914)	1589	94	HCIC	391	1101	97	12	449	1101	27
*Psychomyia flavida* Hagen, 1861	10574	127	HCIC	4291	6070	207	248	1102	6402	2644
RHYACOPHILIDAE (8)										
*Rhyacophila brunnea* Banks, 1911	78	9	HCIC	28	50	0	0	44	19	0
*R. fuscula* (Walker, 1852)	42	14	HCIC	421	1	0	0	0	366	56
***R. glaberrima* Ulmer, 1907**	1	1	INHS	0	1	0	0	0	1	0
*R. ledra* Ross, 1939	1	1	HCIC	0	0	1	0	0	1	0
*R. lobifera* Betten, 1834	5	1	HCIC	0	0	5	0	0	0	5
*R. mainensis* Banks, 1911	29	6	HCIC	3	26	0	0	4	24	1
*R. manistee* Ross, 1939	313	15	HCIC	0	313	0	0	32	244	37
*R. vibox* Milne, 1936	246	12	HCIC	11	233	0	1	236	7	0
SERICOSTOMATIDAE (1)										
*Agarodes distinctus* (Ulmer, 1905)	125	9	HCIC	6	18	101	16	0	5	3
Thremmatidae (3)										
*Neophylax concinnus* McLachlan, 1871	185	32	HCIC	86	92	7	11	45	127	1
*N. fuscus* Banks, 1903	54	4	UMMZ	0	54	0	0	0	54	0
*N. oligius* Ross, 1938	134	18	HCIC	19	80	35	0	62	58	4

**Figure 2.**
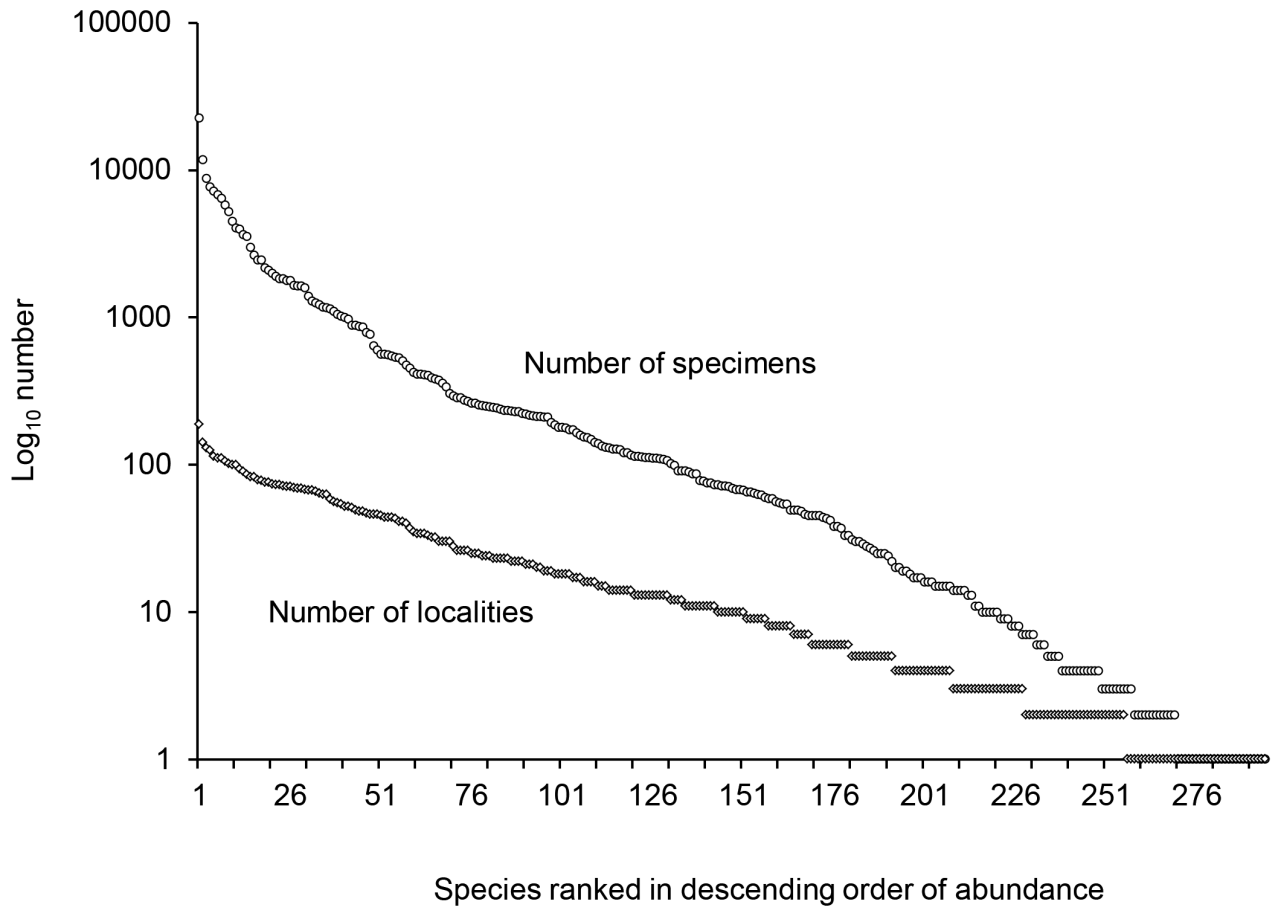
The number of specimens known for each Michigan species and the number of localities where each species has been found.

**Figure 3.**
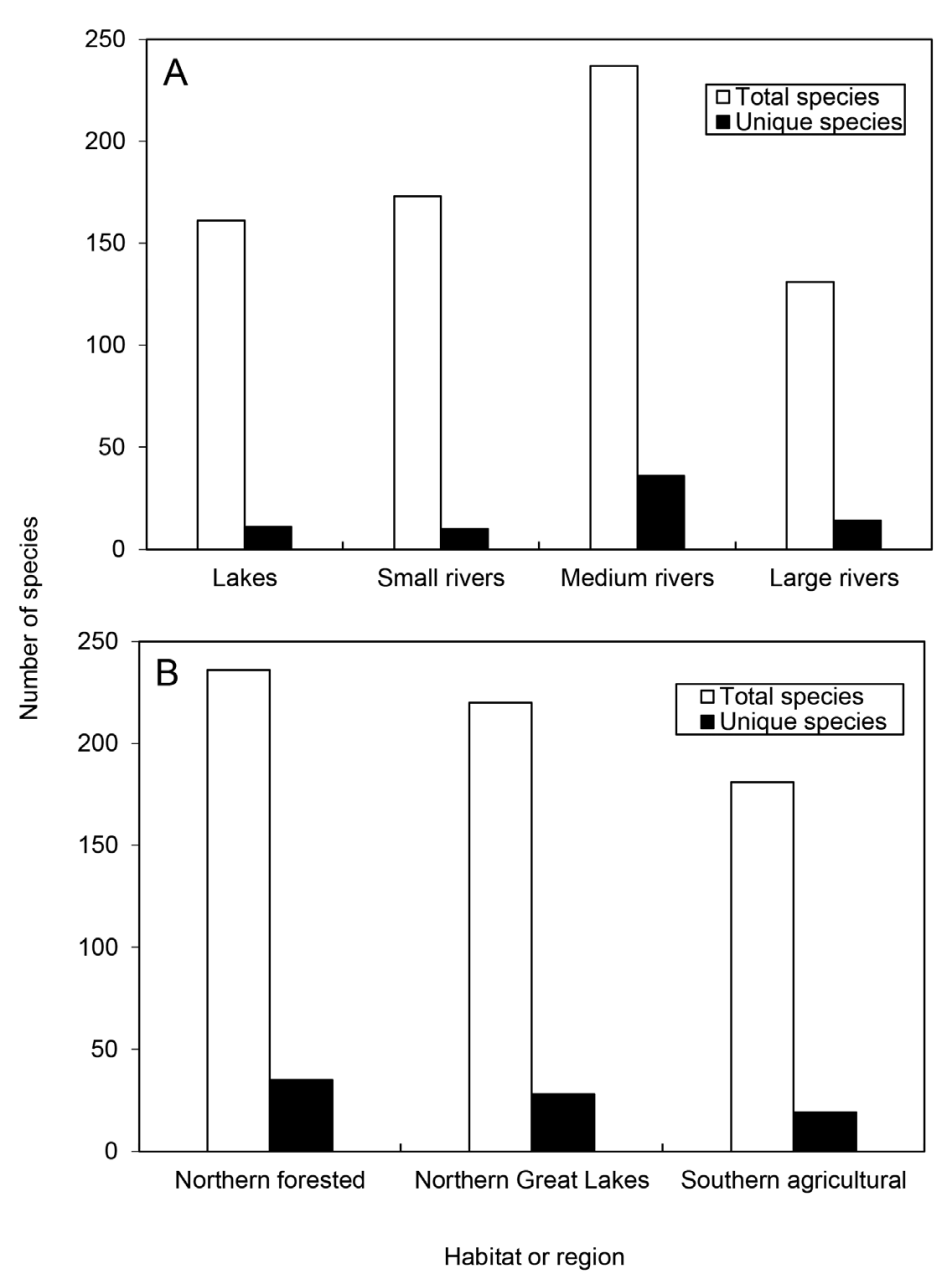
The number of total and unique species from Michigan habitat types (**A**) and caddisfly regions (Houghton 2015) (**B**).

*Oecetis inconspicua* (Walker) (Leptoceridae) was the most widespread species, followed by *Psychomyia flavida* Hagen (Psychomyiidae) and *Helicopsyche borealis* (Hagen) (Helicopsychidae) (Table 2). *Oecetis inconspicua* was also the most abundant species, followed by *P. flavida* and *Chimarra socia* (Hagen) (Philopotamidae). The 11 most abundant species collectively represented over half of all specimens collected. Conversely, 80 species were known from <10 specimens, and 27 species from a single specimen (Figure 2). Hydroptilidae (63 species), Limnephilidae (49), and Leptoceridae (46) were the most species-rich families; *Hydroptila* (28), *Hydropsyche* (21), and *Limnephilus* (20) the most species-rich genera (Table 2). The Northern Forested region contained both the most total species and the most species found exclusively in a single region, followed by the Northern Great Lakes and Southern Agricultural regions (Figure 3). Medium (4–15 m) rivers had the most total and unique species, followed by small (<4 m) streams, lakes, and large (>15 m) rivers.

## Discussion

Nearly all of the species reported here are based on verified adult male specimens. The current location of these specimens is reported in Table 2. One exception is *Rhyacophila lobifera* Betten (Rhyacophilidae), whose presence in Michigan is based on larvae and genetic analysis (Abigail Fusaro, unpublished data). Adult male specimens of *Beothukus complicatus* (Banks) (Phryganeidae) were collected by PLH and identified by BJA. The specimens were subsequently lost. Due to the distinctness of the male genitalia, it is unlikely that these specimens were misidentified and so *B. complicatus* is included on the checklist. Likewise, *Fabria inornata* Banks (Phryganeidae) was included in Leonard and Leonard’s (1949b) checklist. We have not been able to locate specimens, but find it unlikely that this distinctive species was misidentified and so have included it on the checklist. Conversely, *Ceraclea nepha* (Ross) and *C. punctata* (Banks) are more difficult to identify so, in the absence of known specimens, are excluded from the checklist (Table 3).

**Table 3.** Species from Leonard and Leonard’s (1949b) checklist that are considered doubtful due to synonymy, misidentification, or our inability to locate a specimen.

Species	Explanation
*Banksiola selina* Betten, 1944	Junior synonym of *B. crotchi* (Wiggins 1956)
*Ceraclea nepha* (Ross, 1944)	Reported from “Crawford”. No specimen located
*Ceraclea punctata* (Banks, 1894)	Reported from “Crawford”. No specimen located
*Cyrnellus marginalis* (Banks, 1930)	Junior synonym of *C. fraternus* (Flint 1964)
*Dicosmoecus quadrinotatus* (Banks, 1908)	Junior synonym of *Onocosmoecus unicolor* (Wiggins and Richardson 1986)
*Hydropsyche alvata* Denning, 1949	Junior synonym of *H. incommoda* (Korecki 2006)
*Hydropsyche bidens* Ross, 1938	Junior synonym of *H. incommoda* (Korecki 2006)
*Hydropsyche bifida* Banks, 1905	Junior synonym of *H. morosa* (Schefter and Unzicker 1984)
*Hydropsyche orris* Ross, 1938	Junior synonym of *H. incommoda* (Korecki 2006)
*Hydropsyche recurvata* Banks, 1908	Junior synonym of *H. alternans* (Schefter and Wiggins 1986)
*Holocentropus glacialis* Ross, 1938	Misidentified. Is *Plectrocnemia cinerea* (INHS)
*Lepidostoma strophis* Ross, 1938	Junior synonym of *L. cinereum* (Weaver 1988)
*Neophylax autumnus* Vorhies, 1909	Junior synonym of *N. concinnus* (Kimmins and Denning 1951)
*Nyctiophylax uncus* Ross, 1944	Misidentified. Is *N. affinis* (INHS)
*Nyctiophylax vestitus* (Hagen, 1861)	Nomen dubium (Morse 1972)
*Platycentropus plectrus* Ross, 1938	Junior synonym of *P. amicus* (Flint 1966)
*Rhyacophila acropedes* Banks, 1914	Junior synonym of *R. brunnea* (Smith 1984)
*Rhyacophila melita* Ross, 1938	Junior synonym of *R. mainensis* (Smith 1984)

**Figure 4.**
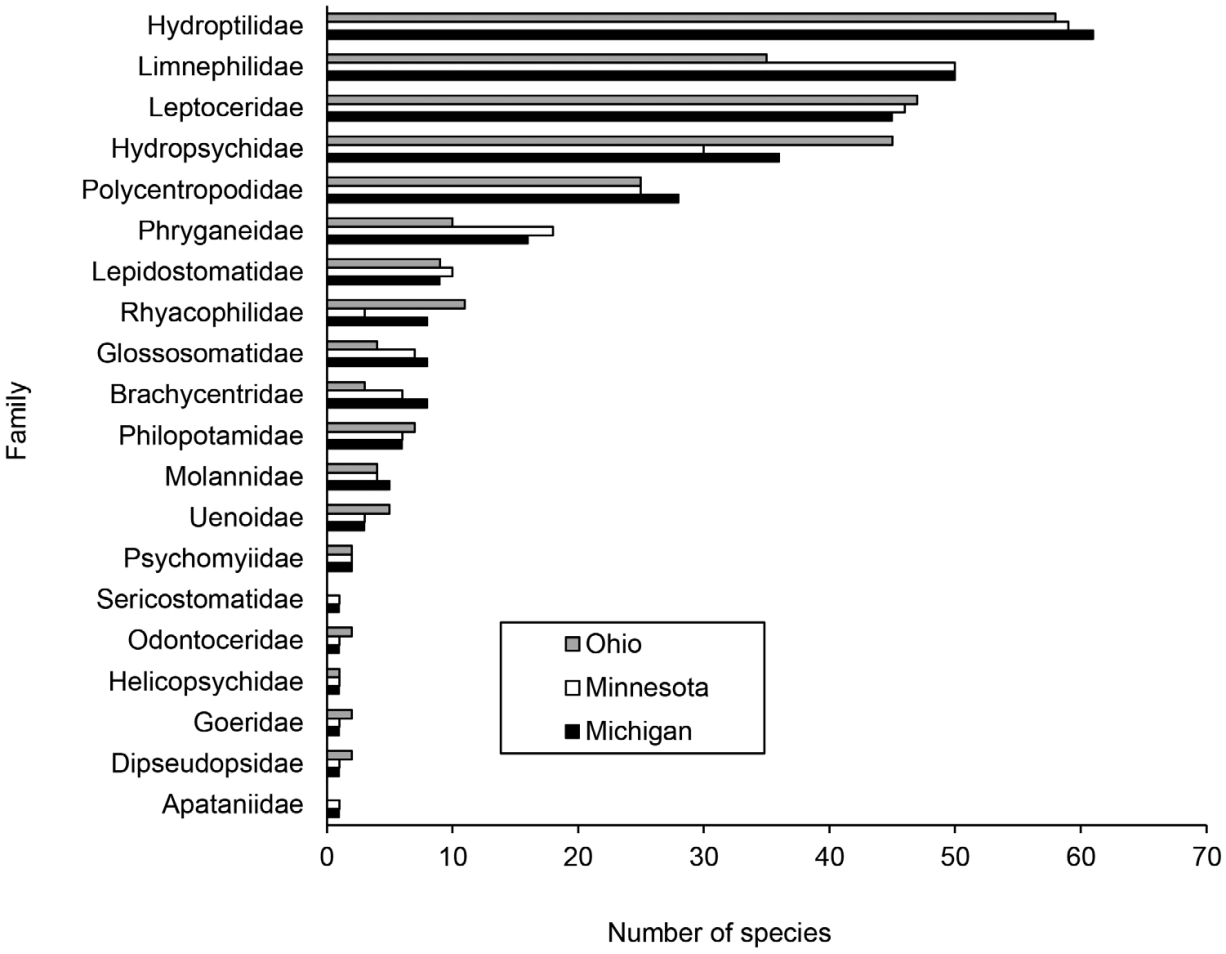
The number of species within families found in Ohio (Armitage et al. 2011), Michigan (present study), and Minnesota (Houghton 2012).

Michigan caddisfly species richness appears similar to that of Minnesota (277 total species) and Ohio (272), two adjacent states where surveys of comparable effort have recently occurred (Armitage et al. 2011, Houghton 2012). All three states generally harbor similar numbers of species in the same families; exceptions include Brachycentridae, Glossosomatidae, Hydropsychidae, Limnephilidae, Phryganeidae, and Rhyacophilidae (Figure 4). Overall distribution of specimens per species (Figure 2) follows a similar pattern in both states (Houghton and Holzenthal 2010).

Michigan and Minnesota also exhibit similar regional patterns, with higher species richness in the Northern Great Lakes and Northern Forested regions than in the Southern Agricultural region (Houghton 2012). These differences are probably due to both natural and anthropogenic factors (Houghton 2015). The majority of streams of the Northern Great Lakes region drain into Lake Superior and are of high gradient, especially their downstream sections. The majority of the region is forested, leading to low levels of watershed disturbance. The Northern Forested region is also relatively undisturbed. Most streams drain into lakes Michigan and Huron and tend to be of lower gradient. Streams of the Southern Agricultural region also tend to be low gradient. The region contains >90% of Michigan’s human population (www.census.gov) and most of its agriculture. Thus, streams are surrounded by anthropogenic disturbance.

Although the majority of Michigan caddisflies have also been collected from adjacent states and provinces, and Michigan does not have any known endemic species, there are still some noteworthy Michigan records reported in this study. *Polycentropus timesis* (Denning) (Polycentropodidae) is known in Michigan from 4 sites in Lake County in the northwestern Lower Peninsula. These sites are separated by >800 km from the other known *P. timesis* collection sites in Massachusetts and New Hampshire (Weaver 1995). Prior to the Michigan collections, *Holocentropus milaca* (Etnier) (Polycentropodidae) and *Oxyethira itascae* Monson and Holzenthal (Hydroptilidae) were both thought to be endemic to Minnesota (Houghton and Holzenthal 2003). *Hydroptila tusculum* Ross (Hydroptilidae) was previously known only from collections in the southeastern U.S. (Moulton and Stewart 1996); the nearest reported collection is ~1,200 km from the single Michigan locality in the northwestern Lower Peninsula. Interestingly, *H. tusculum* has also been collected from Wisconsin (unpublished data), indicating that it is more widespread than originally thought. Similarly, *Neureclipsis piersoni* Frazer and Harris and *Nyctiophylax serratus* Lago and Harris (Polycentropodidae) are known in Michigan from Sleeping Bear Dunes National Park in the northwestern Lower Peninsula. Both species represent >500 km range extensions from their nearest known collecting localities in Kentucky (Rasmussen and Morse 2016).

Including the current study, 20% of the total caddisfly fauna of Michigan, and almost 40% of the hydroptilid fauna, has been reported during the last 10 years, despite a >80-year collecting history in the state (Table 1). Moreover, nearly all recent regional studies have resulted in new state records. Thus, it is likely that additional species remain undiscovered in the state. Future research will include a more comprehensive faunal analysis relating species to habitat preferences and anthropogenic disturbance levels, as well as a conservation assessment of individual Michigan species.

## Supplementary materials

Supplementary material 1 Data file for new state records Data type: speciemens data This is an open access article distributed under the terms of the Creative Commons Attribution License (CC BY 4.0), which permits unrestricted use, distribution, and reproduction in any medium, provided the original author and source are credited. David C. Houghton, R. Edward DeWalt, Angelica J. Pytel, Constance M. Brandin, Sarah E. Rogers, David E. Ruiter, Ethan Bright, Patrick L. Hudson, Brian J. Armitage
